# Adherence to the Mediterranean Diet and its association with gastric cancer: health benefits from a Planeterranean perspective

**DOI:** 10.1186/s12967-024-05176-w

**Published:** 2024-05-21

**Authors:** Claudia Reytor-González, Ana Karina Zambrano, Martha Montalvan, Evelyn Frias-Toral, Alison Simancas-Racines, Daniel Simancas-Racines

**Affiliations:** 1 Centro de Investigación en Salud Pública y Epidemiología Clínica (CISPEC), Facultad de Ciencias de la Salud Eugenio Espejo, Universidad UTE, 170129 Quito, Ecuador; 2 Centro de Investigación Genética y Genómica, Facultad de Ciencias de la Salud Eugenio Espejo, Universidad UTE, Mariana de Jesús Ave, no number, 170129 Quito, Pichincha Ecuador; 3 Universidad Católica Santiago de Guayaquil, . Pdte. Carlos Julio Arosemena Tola, 090615 Guayaquil, Ecuador; 4 School of Medicine, Universidad Católica Santiago de Guayaquil, Guayas, Guayaquil Ecuador; 5 Escuela de Medicina, Universidad Espíritu Santo, 0901952 Samborondón, Ecuador; 6 Carrera de Medicina Veterinaria, Facultad de Ciencias Agropecuarias y Recursos Naturales, Universidad Técnica de Cotopaxi, 050108 Latacunga, Ecuador

**Keywords:** Gastric cancer, Mediterranean diet, Nutrition, Molecular mechanism, Planeterranean perspective

## Abstract

The Mediterranean Diet (MD) has garnered increasing attention for its potential protective effects against gastric cancer (GC). The MD’s rich content of antioxidants, polyphenols, and other bioactive compounds contributes to its ability to modulate gene expression, inhibit tumor growth, and regulate apoptosis. Studies have shown significant reductions in inflammatory markers such as C-reactive protein (CRP), tumor necrosis factor-alpha (TNF-α), and interleukin-6 (IL-6) among individuals adhering to the MD, suggesting its pivotal role in mitigating chronic inflammation-associated with cancer development. Furthermore, the MD’s anti-angiogenic properties, particularly in components like olive oil, red wine, fish, and tomatoes, offer promising avenues for reducing GC risk by inhibiting tumor angiogenesis. Additionally, the MD’s influence on intestinal microbiota composition underscores its potential in maintaining immune homeostasis and reducing systemic inflammation, factors crucial in GC prevention. Despite challenges such as variability in dietary adherence scoring systems and the need for further gender and geographical-specific studies, evidence supports the MD as a cost-effective and holistic approach to GC prevention. Emphasizing the role of nutrition in public health is a promising strategy with broad implications for global health and cancer prevention initiatives. Therefore, this review explores the multifaceted impacts of the MD on GC prevention, delving into its anti-inflammatory, anti-angiogenic, and molecular mechanisms.

## Introduction

According to the World Health Organization (WHO), cancer was the leading cause of death for individuals under 70 years old in over 110 countries in 2019 [1], with approximately 10 million deaths worldwide [2]. Gastric cancer (GC) accounts for 7.7% of all cancer-related deaths and remains a public health concern despite a recent decline in incidence [2, 3]. The incidence rate shows geographic disparities, with the highest rates in East Asia, Eastern Europe, and South America and lower in North America and most of Africa [4].

Approximately 90% of gastric cancers are adenocarcinomas, originating in the mucosal glands in the superficial layer of the stomach [5]. Other types of gastric cancer include mucosa-associated lymphoid tissue lymphomas (MALT) and leiomyosarcomas. Lauren’s classification divides GC adenocarcinomas into diffuse and intestinal types, which differ in microscopic appearance, gender ratio, and age at diagnosis [6]. It is also relevant to highlight the distinction of this neoplasm according to the anatomical subsite of the stomach between cardia GC and non-cardia GC, as they show different epidemiological patterns and causes [5].

This multifactorial disease is related to environmental, genetic, socioeconomic, and lifestyle factors [5, 7, 8], crucial in developing this neoplasm. Among these factors, diet emerges as a component with a considerable impact on the risk of GC. For this reason, nutritional guidelines recommend healthy dietary patterns, including high consumption of cereals, fruits and vegetables, legumes, and white meats and low consumption of red meat, processed food, and those high in fat [9–11].

Several studies have reported that a diet high in sodium and fat can significantly increase the risk of GC due to salt irritation in the gastric wall, which promotes carcinogenesis and the possible increase of Helicobacter Pylori colonization [3, 12–14].

Regarding cereal intake, some studies suggest a reduction in the risk of developing GC by up to 39% in groups with a high intake of this food compared to the group with a lower intake [9, 15, 16].

In the past, Mediterranean countries have reported lower incidences of cancer [17, 18], encouraging the scientific community to explore the possible protective effects of different dietary patterns against this disease.

The Mediterranean Diet (MD), recognized by UNESCO as an intangible cultural heritage, reflects a rich and diverse dietary tradition originating in Mediterranean Sea civilizations [19]. Coined by Ancel Keys in 1960, this dietary pattern has historically been associated with a long and healthy life characterized by low rates of chronic diseases [20]. The MD encompasses a variety of nutritional practices from countries such as Greece, Crete, and southern Italy. However, variants exist elsewhere in Italy, France, Lebanon, Morocco, Portugal, Spain, Tunisia, Turkey, and more, all united by the central use of olive oil, a high consumption of vegetables, fruits, legumes, and grains, with moderate consumption of alcohol, mainly wine, and a low intake of red meat [21].

This dietary pattern is not only appreciated for its nutritional and health benefits [21–23], particularly in the prevention of coronary heart disease and certain forms of cancer, including GC [24], but also for its respect for the environment, promoting sustainable agricultural practices. The Mediterranean culture, which values the consumption of minimally processed, fresh, and locally sourced foods, has integrated ingredients from other regions, adapting them to its mild climate and culinary traditions, further enriching the diet [25].

This review aims to consolidate the current evidence and provide a comprehensive view of the MD’s potential protective effects against gastric cancer. Such exploration not only advances scientific knowledge but also has the potential to influence global dietary patterns, promoting healthier practices with positive health outcomes.

## Methods

For this narrative review, significant publications from 1990 to 2023 were considered. The search was conducted through PubMed and Cochrane Library, using a combination of related search terms, including “Dietary Patterns,” “Mediterranean Diet,” “Diet,” “Gastric Cancer,” “Stomach Cancer,” and “Neoplasms.” Based on their relevance, two research team members (CR-G and DS-R) reviewed the titles and abstracts of the identified articles, selecting them for full review only if both authors agreed. Additionally, the references of the identified articles were analyzed to include these publications. Finally, the selected articles were subjected to a complete content review for evidence of the association between the Mediterranean Diet and gastric cancer.

### Mediterranean Diet and its impact on cancer

Currently, the scientific community’s focus has been directed toward investigating the effects of dietary habits on overall health rather than analyzing specific nutrients [26], as people do not consume these nutrients in isolation, as proposed by the Dietary Guidelines 2020–2025 [27].

Diet quality is related to a lower risk of developing digestive system cancers. Studies indicate that maintaining a rich and balanced diet could significantly decrease the risk of facing these types of cancers. In this regard, the detailed survey of Bertuccio et al. investigated how different eating styles influence the risk of gastric cancer. They found that following a dietary regimen centered on abundant consumption of fruits and vegetables, referred to as “Prudent/Healthy”, is associated with a lower probability of developing gastric cancer (GC). In contrast, an “Western/Unhealthy” diet, characterized by a high intake of starchy foods, meats, and fats, could increase this risk [28].

Adherence to the dietary pattern is also related to decreased risk of different types of cancers. Three case-control studies carried out in Italy between 1992 and 2000 suggest that a 1-point increase in the Mediterranean Diet (MD) score is associated with a 23% reduction in the risk of oropharyngeal cancer, a 28% reduction in esophageal cancer risk, and a 29% reduction in laryngeal cancer risk [29]. Adherence to this dietary pattern is also linked to a lower risk of pancreatic cancer. A systematic review, which included 8 studies conducted in Italy, Singapore, the United States of America (USA), Sweden, and the Netherlands, supports the connection that a healthy, plant-based diet reduces the risk by up to 18%, hile high red meat intake is associated with an increased risk [30]. Research on colorectal cancer suggests that higher dietary scores are associated with an 8–54% lower risk [25]. In breast cancer, one case-control study conducted in the USA found that higher Mediterranean dietary scores were associated with a 24% reduction in the incidence of breast cancer in Hispanic and non-Hispanic American women and a 35% reduction in Asian American women [31, 32]. Moreover, the risk of endometrial cancer can be reduced by more than 50% in women with increased adherence to this eating style [33].

Developing a specific scoring system to assess adherence to the MD [34] and its various adaptations has significantly enhanced the study of this dietary pattern, thereby facilitating research in nutritional epidemiology [34, 35]. This evidence has enabled the assessment of the impact of dietary habits on health. Current evidence strongly supports the protective role of the MD, demonstrating its association with a lower risk of various chronic diseases [21, 36, 37] and the positive effects of this dietary pattern on the incidence and mortality of different types of neoplasms [29, 32, 38–43], highlighting its importance in prevention and health promotion.

However, it’s essential to consider the different criteria for assessing adherence to the MD and variations in the foods consumed by other populations, as this may modify the health effects. Dietary assessments can encompass the combined effect of individual food components, whose health impacts might go unnoticed separately. Additionally, these assessments can overcome nutritional collinearity and reflect the complex biological interactions between different nutritional variables [44].

In the context of using diet as a strategy to prevent cancer or reduce the risk of the disease, angioprevention emerges, promoting the use of substances that inhibit angiogenesis for neoplasm prevention [45]. The antiangiogenic capacity in several natural compounds of certain foods in the MD makes this dietary style particularly attractive as a source of chemopreventive agents within the angiopreventive strategy [46].

Olive oil, a fundamental component of the MD, is recognized for its beneficial health effects. This oil, extracted from olive trees, is classified into various categories according to the production process [47]. Its complex composition includes a saponifiable and an unsaponifiable fraction. The former, primarily composed of triglycerides, contains oleic acid as the primary fatty acid. The unsaponifiable fraction, rich in various compounds such as hydrocarbons, tocopherols, pigments, and phenolic compounds, is responsible for the stability of the oil and contributes to its organoleptic properties [48, 49]. Extra virgin olive oil, in particular, stands out for its high content of phenolic compounds compared to refined oils. Numerous studies have revealed that these compounds have health benefits not solely attributed to oleic acid [50, 51]. Some minority compounds, especially those of the phenolic fraction, also play an essential role.

Certain compounds in olive oil, such as oleuropein, hydroxytyrosol, and triterpenic acids, exhibit antiangiogenic properties, which may decrease cancer risk. Oleuropein, a secoiridoid, acts as a chemopreventive agent by modulating various oncogenic signaling pathways [52]. Its antiangiogenic activity is attributed to the inhibition of VEGFR-2 autophosphorylation and suppression of endothelial cell proliferation and migration [53]. Hydroxytyrosol, derived from oleuropein during olive ripening, exhibits antiangiogenic properties by inhibiting endothelial cell growth and migration [54] and anti-inflammatory and antitumor effects [52, 55]. Triterpenic acids, such as betulinic [56], oleanolic [57], ursolic [58], and maslinic [59] acids, present in virgin olive oil, demonstrate antineoplastic effects and inhibit angiogenesis through various signaling pathways.

Other antiangiogenic compounds in olive oil include flavonoids, carotenoids, vitamin E, diterpenoids, and sterols [45, 60]. These compounds, such as taxifolin [52, 61], β-carotene [62], and lutein [63], have been shown to inhibit the formation of endothelial cell structures and regulate the expression of various angiogenic factors [64].

In addition to olive oil, other components of the MD, such as red wine, contain polyphenols with antiangiogenic properties [65]. Among them, resveratrol [66], piceatannol [67], fisetin [68], delphinidin [69], and myricetin [70] have demonstrated inhibitory effects on angiogenesis, potentially contributing to the health benefits associated with a decreased risk of different malignancies.

Fish and tomatoes may also impact angiogenesis and cancer prevention. Consumption of omega-3 fatty acids in fatty fish is associated with inhibition of angiogenesis [71], and components in tomatoes, such as lycopene and cystine knot miniproteins, also exhibit antiangiogenic properties [72].

The findings highlighted the MD’s significant contribution as a practical nutritional approach to preventing cancer and promoting optimal health. This supports the idea that adopting this dietary pattern can reduce the risk of various chronic diseases, including cancer, and improve quality of life. Promoting and broadening the acceptance of the Mediterranean Diet, considering cultural and regional differences, is an essential strategy in the global fight against cancer and towards a healthier and more sustainable future, as demonstrated.

### Anti-inflammatory effects of the Mediterranean Diet

Western dietary patterns, characterized by high consumption of sugars and saturated animal fats, trigger oxidative stress and inflammation at the cellular level. This leads to adipogenesis and increased production of reactive oxygen species (ROS) [73], increasing the production of proinflammatory biomarkers such as Interleukin 6 (IL-6), tumor necrosis factor-alpha (TNF-α), and Leptin while decreasing the secretion of Adiponectin, an anti-inflammatory hormone [50, 74]. This chronic inflammation contributes to developing diseases such as cancer [21], obesity, dyslipidemia, diabetes, and metabolic syndrome [74], perpetuating a cycle of chronic inflammation that affects the whole organism.

In contrast, the dietary pattern of Mediterranean countries, known for its multiple health benefits [75–78], is characterized by high consumption of extra virgin olive oil, cereals, nuts, fruits, legumes, and white meats; moderate consumption of low-fat dairy products and alcohol; and low consumption of sugars, fats, red meat, and processed foods [79]. These foods are rich in polyphenols, which act as antioxidants, reduce oxidative stress, modulate cell signaling pathways involved in inflammation, regulate the production of proinflammatory cytokines, and influence immune system cells [80, 81].

Observational studies have reported significant inverse associations between MD scores and inflammatory cytokines [82]. While some clinical studies, mainly in non-Mediterranean countries, showed nonsignificant reductions, those conducted in Spain revealed significant decreases in C-reactive protein (CRP) with adherence to MD [83].

Prospective randomized studies have indicated that a low-inflammatory diet, such as MD, is associated with decreased inflammatory biomarkers and improved pain and functionality compared with the usual diet [84]. Also, cross-sectional studies have highlighted significant associations between higher dietary scores and mainly Mediterranean anti-inflammatory and inflammatory markers [85]. Different studies provide evidence of MD’s benefits in reducing inflammation through various approaches.

Polyphenols exert anti-inflammatory effects by modulating several signaling pathways. The nuclear factor kappa B (NF-kB) pathway inhibits NF-kB activation by preventing its release and translocation to the cell nucleus, blocking phosphorylation of inhibitory protein kappa B (IκB), and regulating oxidant levels. These actions prevent the activation of proinflammatory genes, thus reducing the inflammatory response [81]. In addition, these substances affect the mitogen-activated protein kinase (MAPK) pathway in several ways. Specifically, they affect protein signaling cascades such as extracellular signal-regulated kinase 1 and 2 (ERK1/2), c-Jun-activated protein kinase (JNK), and protein kinase 38 (p38). Polyphenols decrease the activation of ERK1/2, JNK, and p38, resulting in reduced TNF release and suppression of proinflammatory cytokine expression [50, 73, 86].

In addition, polyphenols interfere with the arachidonic acid pathway by inhibiting enzymes such as phospholipase A2 (PLA2), cyclooxygenase (COX), and lipooxygenase (LOX), thereby reducing the production of prostaglandins and leukotrienes. These mechanisms contribute to its therapeutic potential in treating chronic inflammatory diseases [87–89].

Another possible explanation for MD’s anti-inflammatory effects is its high fiber content. As these fibers are not digested in the intestine, they are fermented by the intestinal microbiota, forming short-chain fatty acids and potential anti-inflammatory effects. In addition, they decrease intestinal permeability, which positively influences intestinal health by creating an environment less prone to developing chronic inflammatory processes, known drivers of gastric cancer [80, 90].

Evidence suggests that the Mediterranean Diet stands out for its benefits against chronic diseases, especially cancer, thanks to its anti-inflammatory effects. Rich in polyphenols and fiber, this dietary pattern fights inflammation and oxidative stress, regulating cellular processes and gene expression and producing proinflammatory biomarkers. Therefore, choosing the Mediterranean Diet could be an effective nutritional strategy for preventing inflammation and its pathological consequences. (Table 1)

**Table 1:** Bioactive compounds in the Mediterranean Diet: sources and mechanisms of anticancer action

Compounds	Sources	Mechanism of Action
Oleuropein Hydroxytyrosol Tyrosol	Extra Virgin Olive Oil Fruits Vegetables Red wine	Modulation of gene expression. Inhibition of cell proliferation. Induction of apoptosis. Inhibition of angiogenesis and metastasis [52–54].
Taxifolin, Quercetin, Catechins	Green tea Apples Citrus Onions	Reduction of oxidative stress. Inhibition of cancer cell formation. Reduction of inflammation [52, 61].
Resveratrol, Piceatannol, Fisetin, Delphinidin and Myricetin	Grapes Red wine	Inhibition of cell proliferation. Induction of apoptosis. Modulation of immune response [65–70].
Ellagic acid	Red fruits Nuts	Prevention of DNA damage. Induction of apoptosis. Inhibition of cell proliferation [86, 91, 92].
Triterpenic Acids (Betulinic, Oleanolic, Ursolic, Maslinic Acid)	Extra Virgin Olive Oil	Inhibition of cell proliferation and angiogenesis. Induction of apoptosis. Modulation of signaling pathways [56–59].
Vitamin E	Extra Virgin Olive Oil Nuts Seeds	Protection of cell membranes from oxidative damage. Modulation of cellular signaling and gene expression related to apoptosis [45, 60].
Carotenoids (Lutein, β-carotene)	Carrots Spinach Tomatoes	Protection against oxidative damage. Modulation of immune function. Tumor growth inhibition [62–64].
Dietary fiber	Whole grains Legumes Fruits Vegetables	Promotion of gastrointestinal health. Reduction of systemic inflammation. Modulation of the intestinal microbiome [80, 90].
Omega-3 fatty acids	Fatty fish Linseed oil	Reduction of inflammation. Inhibition of tumor angiogenesis. Modulation of apoptosis [71].

### The protective role of the Mediterranean Diet in mitigating gastric cancer risk

The impact of diet as a risk factor for each subtype of gastric adenocarcinoma (cardia and non-cardia) remains unclear. However, scientific evidence highlights that a dietary pattern high in salt and processed foods increase the risk of gastric cancer (GC) by up to 22% [3, 93–95], while consuming fruits and vegetables could exert a preventive effect [96].

In this context, the MD, known for its emphasis on whole grains, vegetables, seafood, and fruits, emerges as a crucial dietary pattern in decreasing the risk of some neoplasms as GC. Studies suggested an inverse relationship between adherence to the MD and the incidence of GC [97], although others have pointed out some discrepancies in the results [93].

The MD, characterized by its rich content of antioxidants such as vitamins E and C, polyphenols, and folic acid, could play a protective role by modulating cancer-related biological pathways [98, 99]. This notion is supported by the findings from the European Prospective Investigation into Cancer and Nutrition (EPIC) study, which highlights the inflammatory effects of unhealthy dietary patterns and their association with an increased risk of gastric cancer. The study also indicates a 33% risk reduction for distal gastric adenocarcinoma and a 55% reduction for gastric adenocarcinoma with increasing adherence to the MD [100–102].

Regarding histological subtypes of GC, research suggests that each 1-unit increase in the MD adherence score was associated with a 7% reduction in risk for intestinal and diffuse types [102].

Despite promising evidence, the field faces challenges, including variability in the dietary adherence scoring systems used [101] and the necessity for more extensive studies to address potential differences by gender [101, 103] and geographic location [97], given the highly influential genetic and environmental component of this neoplasm.

### Exploring molecular mechanisms underlying the Mediterranean Diet’s protective effect against gastric cancer

Research on the molecular mechanisms underlying the protective effect of MD against gastric cancer has revealed a complex interconnection of interacting factors at the genetic and cellular levels [104]. However, the precise molecular mechanism by which this diet exerts protective effects against cancer is still under investigation. It is difficult to isolate the effects of all the bioactive compounds in the foods that characterize this dietary pattern. Several studies have reported that the combined effects of these bioactive substances prevent the progression of various malignancies, including gastric cancer [92, 104]. A crucial aspect focuses on the ability of the MD to modulate gene expression, specifically the regulation of tumor suppressor genes and the inhibition of tumor promoter genes [104].

Specific components of the MD, such as the polyphenols present in extra virgin olive oil, positively influence well-known tumor suppressor genes, including the p53 and p21 proteins [91], crucial in inhibiting tumor growth by regulating apoptosis and cell cycle arrest [105–108].

The MD’s influence on the molecular signaling cascade related to apoptosis, a programmed cell death process crucial for cancer prevention, has been further explored [109]. Specific dietary components, such as flavonoids in fruits and vegetables, exhibit proapoptotic properties by interacting with essential proteins in the apoptotic signaling pathway. These interactions may trigger the selective elimination of potentially cancerous cells, thus contributing to the MD’s antineoplastic effect in gastric neoplasms [110, 111].

Research on how the MD affects vital molecules such as C-reactive protein (CRP), tumor necrosis factor-alpha (TNF-α), and interleukin-6 (IL-6) has deepened [80, 81]. Chronic low-grade inflammation caused by inadequate diet increases cancer risk by promoting initial genetic mutation, progression, and metastasis [112]. Studies have indicated significant reductions in these inflammatory markers in individuals following the MD [73, 75], reinforcing the hypothesis that the anti-inflammatory capacity of this diet may be one of the fundamental pillars of its preventive effect against gastric cancer.

### The planeterranean perspective of the Mediterranean Diet in gastric cancer prevention

Adopting the MD, widely recognized for its effectiveness in preventing chronic diseases, including gastric cancer [113], opens new horizons from a “Planeterranean” perspective, a term proposed by the UNESCO Chair on Health Education and Sustainable Development for sustainable dietary models [114]. This approach encourages the local adaptation of MD’s nutritional principles and focuses on sustainability and using locally available foods [25].

This perspective may have significant implications for the prevention of GC. MD is rich in fruits, vegetables, olive oil, and whole grains and contains essential elements such as antioxidants, phenolic compounds, and dietary fiber that prevent the development of cancer cells in the stomach [115]. Introducing a Planeterranean perspective facilitates the adaptation of these healthy dietary principles to locally available foods, allowing their use in different geographic regions, regardless of their traditional location of the MD and favoring preventive effects against GC.

Furthermore, reducing the consumption of red and processed meats is an essential factor in preventing stomach cancer and is a crucial recommendation of the MD [116]. By increasing the intake of plants and locally available plant foods, the Planeterranean perspective can help minimize the consumption of red and processed meats, thus directly addressing one of the risk factors for GC.

Sustainability, a pillar of the Planeterranean vision, emphasizes choosing foods for their health benefits and to minimize their impact on the planet. Sustainable nutritional approaches can help prevent stomach cancer by promoting diets emphasizing a healthier environment and responsible consumption.

In this context, current regional eating habits are analyzed, and possibilities are identified for integrating the principles of the MD by adapting them to local food availability [117]. Regional recommendations, ranging from the consumption of canola oil, pecan nuts, and okra in North America to avocado and quinoa in Latin America [118, 119], point to a shift towards healthier and more sustainable diets. In Africa, the consumption of indigenous grains such as teff and moringa is emphasized, while sesame oil and marine macroalgae are recommended in Asia [120, 121]. On the other hand, Australia benefits from using local nuts and fish rich in omega-3 fatty acids [113].

The Planeterranean Perspective provides a comprehensive and adaptive vision of the MD, not only as a nutritional strategy for preventing gastric cancer but also as a sustainable model for global well-being and planetary health, emphasizing its relevance.

## Conclusions

This review offers a detailed analysis of the MD’s protective benefits against gastric cancer, emphasizing its significance in reducing the risk of this disease. A thorough scientific literature search shows that the MD has significant anti-inflammatory, anti-angiogenic, and molecular impacts.

The MD anti-inflammatory effects could help counter chronic inflammation linked to cancer development. The foods comprising this diet contain polyphenols that can alter various signaling pathways, thereby reducing the production of inflammatory biomarkers. These findings underscore the crucial role of this MD in preventing gastric cancer while also suggesting other health benefits, such as weight loss and improvement in pain and physical function biomarkers.

Furthermore, this study highlights the relationship between the MD and the prevention of angiogenesis, a crucial process for tumor growth. Olive oil, red wine, fish, and tomatoes contain anti-angiogenic compounds that can help reduce the risk of GC. These findings emphasize the significance of considering our diet as a nutritional element and a potential tool for broader cancer prevention strategies.

The MD regulates gene expression at the molecular level, particularly of tumor suppressor genes like p53 and p21. Olive oil, rich in polyphenols, positively influences these pathways, suppressing gastric cancer cell formation and proliferation. The diet also regulates apoptosis and intestinal microbiota composition, suggesting a protective role in cancer prevention. Therefore, the MD could prove to be a more cost-effective cancer prevention strategy and reduce the toxicity associated with chemotherapy.

Additionally, the Planeterranean perspective redefines the application of the MD beyond its traditional boundaries. It promotes the local and sustainable adaptation of the MD’s nutritional principles to fight gastric cancer and promote global wellness. This approach emphasizes the importance of incorporating local ingredients into the diet, reducing consumption of red and processed meats, prioritizing sustainability, and consuming foods that respect human health and the environment, demonstrating that these dietary practices can effectively prevent gastric cancer.

While the research findings are encouraging, some challenges remain to overcome. One of the challenges is the variability of dietary adherence scoring systems. In addition, more extensive studies are needed to address possible gender and geographical differences. Despite these challenges, the evidence supports adopting Mediterranean dietary patterns as a promising strategy for preventing GC. This highlights the importance of nutrition in public health. This analysis advances scientific knowledge and promotes healthy nutritional practices that positively affect global health.
